# PD-L1 Over-Expression Varies in Different Subtypes of Lung Cancer: Will This Affect Future Therapies?

**DOI:** 10.3390/clinpract12050068

**Published:** 2022-08-24

**Authors:** Asad Ullah, Steven Pulliam, Nabin Raj Karki, Jaffar Khan, Sana Jogezai, Sandresh Sultan, Lal Muhammad, Marjan Khan, Nimra Jamil, Abdul Waheed, Sami Belakhlef, Intisar Ghleilib, Eric Vail, Saleh Heneidi, Nagla Abdel Karim

**Affiliations:** 1 Department of Pathology, Vanderbilt University Medical Center, Nashville, TN 37232, USA; 2 Medical College of Georgia, Augusta, GA 30912, USA; 3 Hematology-Oncology/Georgia Cancer Center, Augusta University, Augusta, GA 30912, USA; 4 Department of Pathology, Indiana School of Medicine, Indianapolis, IN 46202, USA; 5 Bolan Medical College, Quetta 83700, Pakistan; 6 Pakistan Institute of Medical Sciences, Islamabad 44000, Pakistan; 7 Marshfiled Clinic, Marshfield, WI 54404, USA; 8 San Joaquin General Hospital, San Joaquin, CA 93660, USA; 9 Department of Pathology and Laboratory Medicine, Cedars-Sinai Medical Center, Los Angeles, CA 90048, USA; 10 Inova Schar Cancer Center, University of Virginia, Fairfax, VA 22031, USA

**Keywords:** programmed death-ligand, immunosuppression, autoimmunity, adenocarcinoma

## Abstract

Programmed death-ligand (PD-L) 1 and 2 are ligands of programmed cell death 1 (PD-1) receptor. They are members of the B7/CD28 ligand-receptor family and the most investigated inhibitory immune checkpoints at present. PD-L1 is the main effector in PD-1-reliant immunosuppression, as the PD-1/PD-L pathway is a key regulator for T-cell activation. Activation of T-cells warrants the upregulation of PD-1 and production of cytokines which also upregulate PD-L1 expression, creating a positive feedback mechanism that has an important role in the prevention of tissue destruction and development of autoimmunity. In the context of inadequate immune response, the prolonged antigen stimulation leads to chronic PD-1 upregulation and T-cell exhaustion. In lung cancer patients, PD-L1 expression levels have been of special interest since patients with non-small cell lung cancer (NSCLC) demonstrate higher levels of expression and tend to respond more favorably to the evolving PD-1 and PD-L1 inhibitors. The Food and Drug Administration (FDA) has approved the PD-1 inhibitor, pembrolizumab, alone as front-line single-agent therapy instead of chemotherapy in patients with NSCLC and PD-L1 ≥1% expression and chemoimmunotherapy regimens are available for lower stage disease. The National Comprehensive Cancer Network (NCCN) guidelines also delineate treatment by low and high expression of PD-L1 in NSCLC. Thus, studying PD-L1 overexpression levels in the different histological subtypes of lung cancer can affect our approach to treating these patients. There is an evolving role of immunotherapy in the other sub-types of lung cancer, especially small cell lung cancer (SCLC). In addition, within the NSCLC category, squamous cell carcinomas and non-G12C KRAS mutant NSCLC have no specific targetable therapies to date. Therefore, assessment of the PD-L1 expression level among these subtypes of lung cancer is required, since lung cancer is one of the few malignances wherein PD-L1 expression levels is so crucial in determining the role of immunotherapy. In this study, we compared PD-L1 expression in lung cancer according to the histological subtype of the tumor.

## 1. Introduction

PD-L1 and 2, which are ligands of the PD-1 receptor, are members of the B7/CD28 ligand-receptor family. They are the most investigated inhibitory immune checkpoints at present [1]. PD-1 receptor (also known as CD279) is a transmembrane protein and a co-inhibitory receptor present on the surface of T- and B-cells, monocytes, as well as activated natural killer cells [2,3]. Naturally, it interacts with two ligands expressed by antigen-presenting cells (APCs), PD-L1 (B7-H1, CD274) and PD-L2 (B7-DC, CD273) [4,5] where PD-L1 is considered to be the main effector in PD-1 reliant immunosuppression [6]. The expression pattern of PD-L1 and PD-L2 is different in that PD-L1 is mainly expressed on T and B cells, macrophages, and dendritic cells, while PD-L2 is expressed on APCs and T-helper cells [7,8,9]. There is also a difference in the mechanism of action of the two ligands; PD-L1 interacts with PD-1 and CD80, whereas PD-L2 interacts directly with PD-1 [5]. The PD-1/PD-L pathway is a key regulator for T-cell activation [10,11,12]. Activation of T-cells warrants the upregulation of PD-1 and production of cytokines which also upregulate PD-L1 expression. This positive feedback mechanism plays an important role in the prevention of tissue destruction and the development of autoimmunity [13,14]. With the inadequate immune response, the prolonged antigen stimulation leads to chronic PD-1 upregulation and T cell exhaustion.

Involvement of the PD-1/PD-L1 pathway in cancer has been established in various solid and hematological malignancies [6]. The upregulation of PD-1 levels on tumor-infiltrating lymphocytes (TILs), when compared to T-cells in peripheral blood or healthy tissues of the patients, is thought to be why TILs have impaired antitumor activity [7]. PD-1 positive TILs have shown diminished T-cell receptor (TCR) signaling, sub-standard calcium flux, and decreased cytokine production [15,16,17,18]. PD-L1, which is upregulated on the surface of tumor cells, intra-tumor macrophages, and APCs, may also have an anti-apoptotic function in cancer cells. One explanation is that its increased expression is highly associated with tumorigenesis and invasion in vivo and resistance to T-cell mediated lysis in vitro [1]. Upregulation of the ligand results from stimulation by proinflammatory cytokines including IFN-γ which is produced by lymphocytes already existing in the tumor microenvironment [19] providing another association between PD-1/PD-L1 immune checkpoint pathway activation and cancer.

PD-L1 expression levels have been of special interest since patients with non-small cell lung cancers (NSCLC) who demonstrate higher levels of expression tend to respond more favorably to the evolving PD-1 and PD-L1 inhibitors [20,21,22]. Since the food and drug administration (FDA) has approved the PD-1 inhibitor pembrolizumab alone as front-line single-agent therapy instead of chemotherapy in patients with NSCLC without an actionable driver mutation (ALK or EGFR) and expressing [PD-L1 Tumor Proportion Score (TPS) ≥ 1%], the study of PD-L1 expression levels in the different subtypes of lung cancer has been of major interest. While NSCLC comprises the majority of lung cancer, other types of lung cancer such as SCLC and large cell neuroendocrine cancer (LCNEC) are both aggressive and understudied in terms of PD-L1 expression levels, with contradictory reports of expression status [23]. In one retrospective study, the expression of PD-L1 >1% was lower than that seen in NSCLC, though stromal cells showed 18.5% positivity [24]. In contrast, a second study found 82.8% of SCLC with positive staining in >5% of tumor cells [25]. Also, within the NSCLC category, squamous cell carcinomas and non-G12C KRAS mutant adenocarcinomas, and other less common categories of NSCLC have no specific targetable therapies to date [26,27,28,29,30,31]. therefore, assessment of the PD-L1 expression level among these subtypes of lung cancer is warranted, since lung cancer is among those malignances wherein PD-L1 expression level is consulted in determining to define role of immunotherapy.

Understanding of these principles and properties has led to many advances in the development of immune checkpoint inhibitors, especially drugs that target PD-1 and PD-L1, and there are now various PD-1/PD-L1 inhibitors that are either approved for the treatment of NSCLC or at different phases of drug development. The leading PD-1 inhibitors are pembrolizumab, nivolumab and cemiplimab, humanized IgG4 isotype monoclonal antibody [32,33], while prominent PD-L1 inhibitors in clinical use, namely atezolizumab, durvalumab, and avelumab are fully humanized IgG1 isotype monoclonal antibodies [34]. The mode of action of the anti-PD-1 IgG4 monoclonal antibodies includes binding to C1q and activating the complement pathway, whereas that of PD-L1 inhibitors includes induction of antibody-dependent cell-mediated cytotoxicity (ADCC) and complement-dependent cytotoxicity through the Fc region of IgG1. Till date, ipilimumab (IgG1 monoclonal antibody cytotoxic T-lymphocyte associated antigen 4, CTLA-4), pembrolizumab, nivolumab, cemiplimab, durvalumab and atezolizumab are approved for various indications in lung cancers.

Immunohistochemistry has been used to evaluate PD-L1 expression as a predictive biomarker. The different drugs in clinical development have been developed with their diagnostic immunohistochemistry test with variations in cut-off values for regarding a sample as positive. Pembrolizumab uses the PD-L1 IHC 22C3 pharmDx (Dako) assay to test membranous staining of PD-L1 on tumor cells, with TPS <1% scored as no expression, 1–49% as low expression, and ≥50% as a high expression [35]. Nivolumab, which also tests membranous staining of PD-L1 on tumor cells, uses the PD-L1 IHC 28-8 pharmDx (Dako) assay on patients with non-squamous histology. Samples are categorized into negative (<1%), low expression (1–5%), intermediate expression (5–10%), or high expression (≥10%) [36]. Durvalumab tests membranous staining of PD-L1 on tumor cells as the method for scoring, using the SP263 antibody assay (Ventana), with samples considered positive if ≥25% of tumor cells express PD-L1 [37].

Atezolizumab uses the SP142 antibody assay (Ventana). PD-L1 expression scores are determined by immunohistochemistry in tumor cells (TC; percentage of PD-L1-expressing tumor cells: TC0 < 1%, TC1 1–5%, TC2 5–50%, and TC3 ≥ 50%) and tumor-infiltrating immune cells (IC; percentage of tumor area: IC0 < 1%, IC1 1–5%, IC2 5–10%, and IC3 ≥ 10%). Patients are considered as being positive with TC1/2/3 and/or IC1/2/3 [38,39].

### Study Aims

1. To assess the PD-L1 expression level in NSCLC, KRAS mutant adenocarcinoma, adenocarcinoma, and squamous cell carcinoma.
2. To assess the PD-L1 expression level in SCLC and large cell neuroendocrine cancer (LCNEC).
3. Role of PD-L1 positive and PD-L1 negative and effect on treatment.

## 2. Methods

### 2.1. Search Strategy

We searched the PubMed database using the keywords “PD-L1 and Squamous Cell Lung Cancer”, and “PD-L1 and Small Cell Lung Cancer”. We searched for articles in English, from 1 January 2010 to 30 June 2017. For “PD-L1 and Small Cell Lung Cancer”, the additional filter of “Human” was used.

### 2.2. Exclusion Criteria

Articles excluded: reviews, meta-analyses, abstract-only, and case reports; articles with duplicate information; studies with insufficient or unusable data; articles not in English.

### 2.3. Data Review

We examined the articles which had not been excluded for study type or duplication of data for information on PD-L1 expression in lung cancer.

## 3. Results

**Figure 1:**
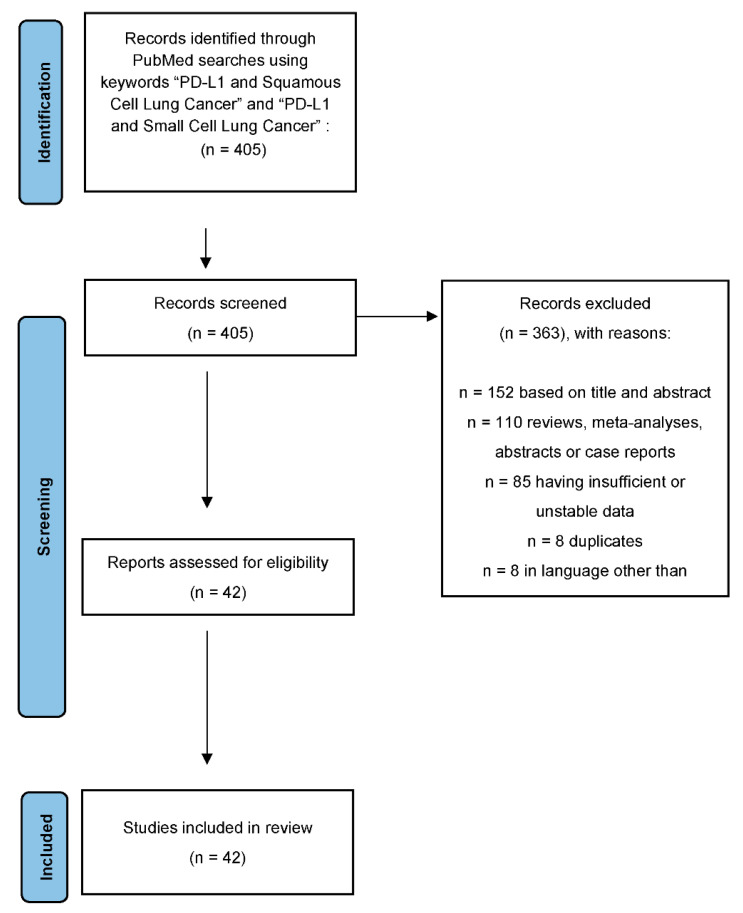
Consort diagram of the study process using Prisma protocol. Note: cut-off of 1–49% includes studies that used unique criteria to determine positivity, such as >5% staining or minimum moderate intensity staining.

A total of 405 results were identified. We excluded 110 reviews, meta-analyses, abstracts or case reports, 8 were duplicates, 8 were in a language other than English, 152 were excluded based upon title or abstract, and 85 had insufficient or unusable data. Thus, 42 studies were included in our analysis (Figure 1).

When levels in NSCLC were reported without further mention of NSCLC subtypes, the positive expression levels of PD-L1 using cut-off values of >1% and ≥50% was 37.03% and 13.29%, respectively. At both the >1% and >5% cut-offs, PD-L1 expression was found to be higher in squamous cell carcinoma than in adenocarcinoma, with values of 41.05% versus 34.72%, and 16.08% versus 9.33% (Table 1).

Limited data were available for SCLC, LCNEC, and KRAS mutant adenocarcinoma. Notably, when using cut-off values between 1–49%, KRAS mutant adenocarcinoma and SCLC both had positive PD-L1 expression in at least 25% of cases (Table 1).

### PD-L1 Clones and Hematoxylin and Eosin (H&E) Staining of Different Types of Lung Cancer

Due to the pervasive involvement of PD-L1 in a variety of cancers, multiple novel avenues have been investigated to target this protein, including antibody blockade, gene silencing, and small molecule inhibition. Commercially available PD-L1 antibodies have shown great success in treating cases of NSCLC and thus have attracted the attention of clinicians and researchers alike [45]. These antibodies work by binding to PD-L1 on the surface of either the tumor cells or antigen-presenting cells, effectively reversing the negative immune regulation induced by this protein [46]. The benefits of antibody therapies as shown in clinical trials include a high objective response rate, prolonged survival and lower rates of adverse events [47,48].

Despite these impressive findings, the efficacy of antibody-based therapy inherently relies on the degree of expression of PD-L1 in patient cells, particularly the membrane (mPD-L1) and serum (sPD-L1) variants. Studies have shown decreased response to antibody therapy in those patients that are negative for PD-L1, further emphasizing the importance of staining for PD-L1 to create patient-centered, targeted therapy [49]. Various antibody clones have been developed, with each showing a different affinity for PD-L1 staining based on its molecular structure.

Kinstler and colleagues analyzed several commercially available antibody clones and their staining profiles for both variants of NSCLC, adenocarcinoma, and squamous cell carcinoma (SCC). Adenocarcinoma, overall, showed greater levels of PD-L1 staining with intermediate expression levels ranging from 43.3–49.7% with clones 405.9A11, E1L3N, 22C3, and SP142, and high expression ranging from 62.2–69.3% with clones 28-8 and SP263. Meanwhile, the only clone to show expression levels above the low range in cases of SCC was SP263 at 44.9% [50]. Given these findings, SP263 appears to be a viable antibody clone that could be used to test for PD-L1 expression in patient tissues with great efficacy. However, clone cost and other confounding factors such as antibody cross-reactivity should be taken into consideration when implementing these tests to guide therapies and further investigations are needed to determine the ideal antibody clone.

Institutions use one of the above clones for PD-L1 expressions. Each clone should be validated and then used for PD-L1. In this study, we used “PD-L1 (22C3) * FDA (Keytruda^®^) for NSCLC” after validation.

**Figure 2:**
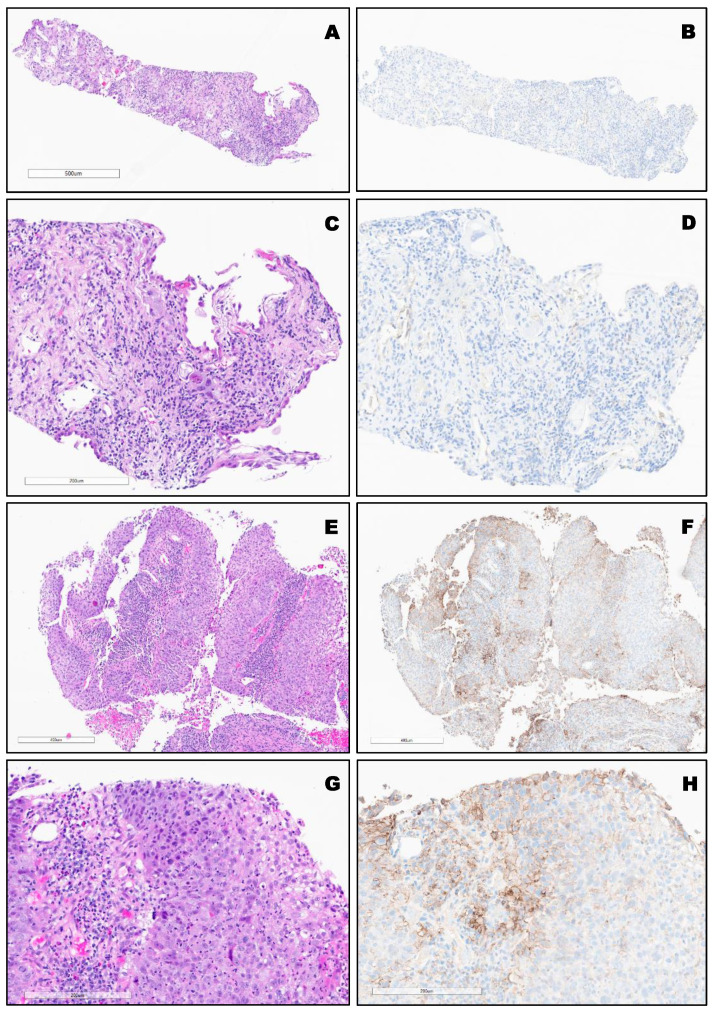
(**A**–**D**), Top SCC (TPS:0%). Bottom, (**E**–**H**): Moderately differentiated non-keratinizing SCC (TPS: 40%) Bottom.

**Figure 3:**
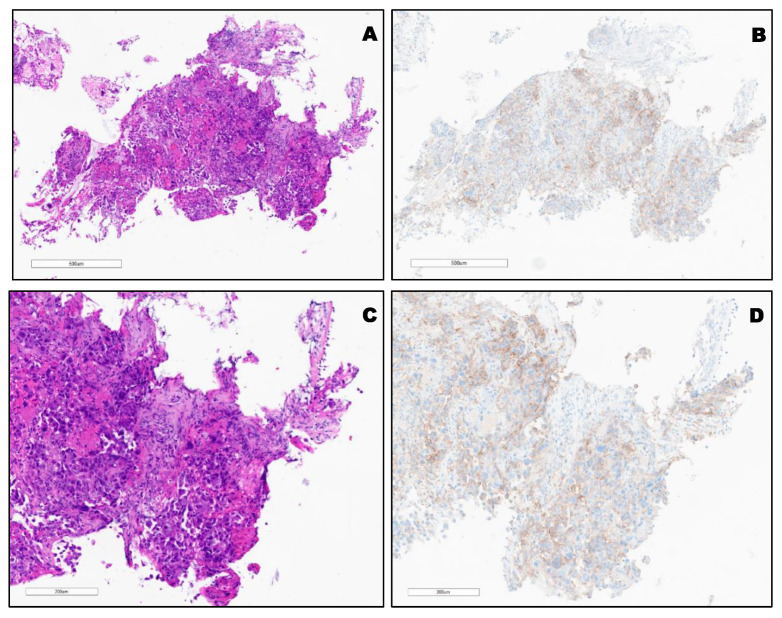
(**A**,**C**) H&E of KRAS mutated (G12C) poorly differentiated SCC. (**B**,**D**), TPS: 30% using PD-L1 (22C3) FDA (Keytruda^®^).

**Figure 4:**
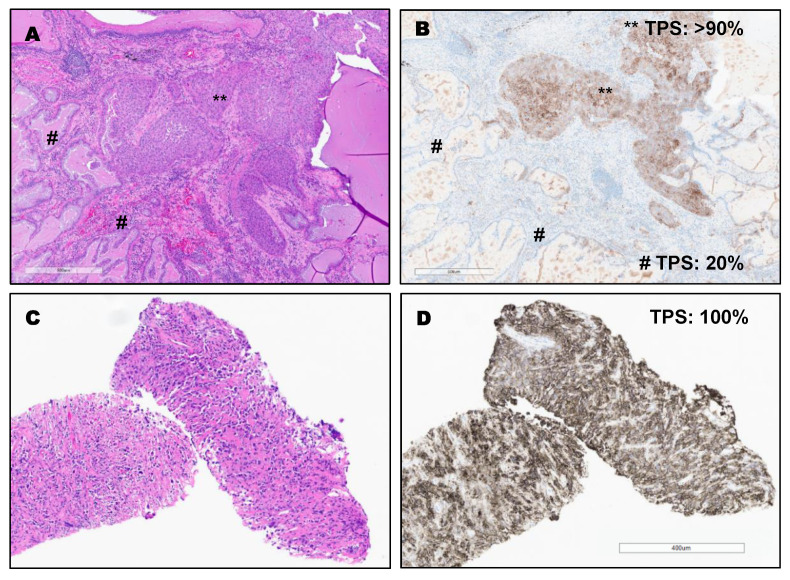
(**A**,**B**): Invasive adenosquamous carcinoma with high expression in squamous carcinoma component (TPS: >90%) and intermediate expression in the adenocarcinoma component (TPS: 20%) by using PD-L1 (22C3) FDA (Keytruda^®^). ** TPS: >90% (squamous carcinoma component). # TPS: 20% (adenocarcinoma component). Bottom (**C**,**D**): Sarcomatoid/Pleomorphic carcinoma with diffuse PDL1 staining (TPS: 100%) by using PD-L1 (22C3) FDA (Keytruda^®^).

**Figure 5:**
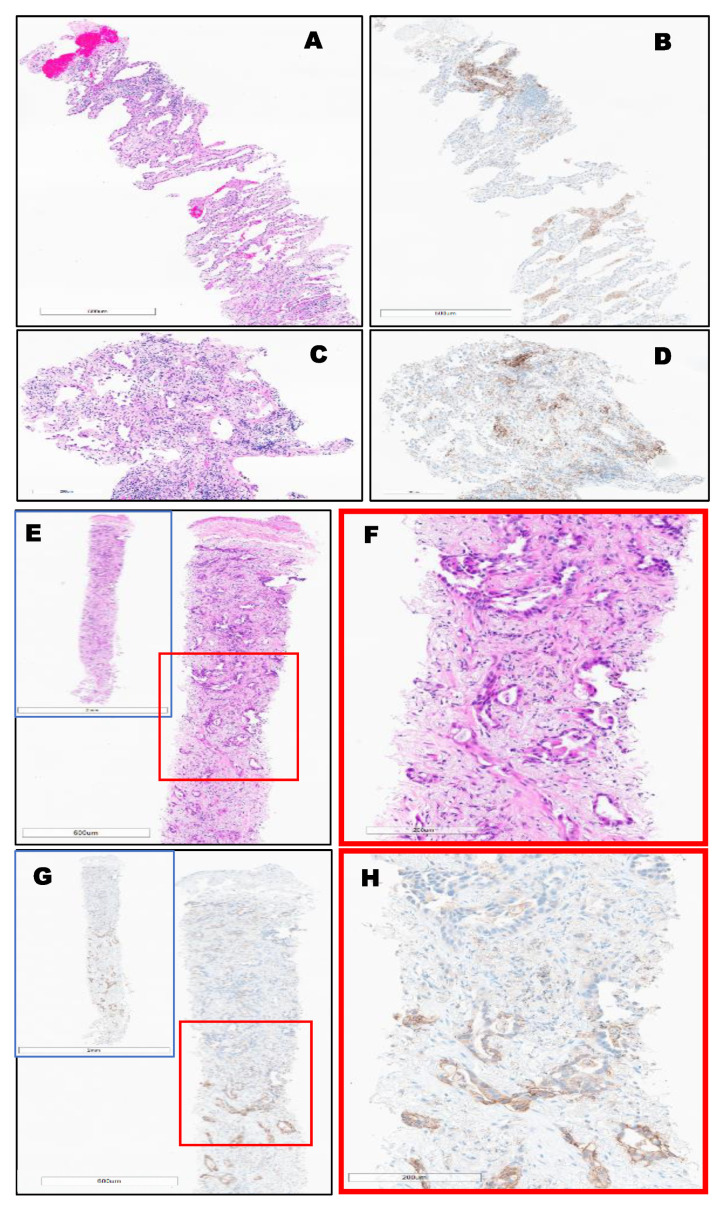
Invasive lung-primary adenocarcinoma, EGFR mutated, (TPS = 5%) Top (**A**–**D**). Bottom, (**E**–**H**): Invasive lung-primary adenocarcinoma identified throughout the entire biopsy, (TPS = 50%) using PD-L1 (22C3) FDA (Keytruda^®^), where a strong stain is only observed in the bottom half of the biopsy (G-H) and not adenocarcinoma at the top of the image.

PD-L1 expression in different types of lung cancer. The variable expression of PD-L1 is shown in Figure 2, Figure 3, Figure 4 and Figure 5.

## 4. Discussion

PD-1 and PD-L1 immune checkpoint inhibitors, effective in the treatment of lung cancer are now approved and/or used for all settings viz neoadjuvant, adjuvant and metastatic either alone or in combination with chemotherapy. A notable example is pembrolizumab, which has been approved either as single-agent therapy instead of chemotherapy for patients with metastatic or aggressive NSCLC and ≥1% PD-L1 expression level without other actionable driver alterations (i.e., ALK, EGFR, NTRK), or in combination with chemotherapy in patients with non-squamous NSCLC for lower stage disease. Immunotherapy as a first line option is to be avoided when the tumor harbors some driver mutations, most notably EGFR activating alterations, due to inefficacy and heightened toxicity. Thus, the assessment of PD-L1 status in lung cancer has been of significant interest and supported by several studies which demonstrate that cancers with ≥50% PD-L1 expression respond very well to PD-1/PD-l1 targeted therapies [20,21,22,40].

It has been previously reported that between 23–28% of patients with advanced NSCLC express high levels of PD-L1, defined as PD-L1 membrane expression on ≥50% of tumor cells [20,21]. More recently, a study of 982 PD-L1 evaluable NSCLC patients showed that 14.3% had tumor cell membrane expression ≥50%, while another study of 1653 stage IV NSCLC patients, found that only 30.2% of patients met this cut-off [44,51]. Importantly, KEYNOTE-001 and KEYNOTE-010 have shown that patients with advanced NSCLC who demonstrate PD-L1 expression ≥50% are more likely to respond to treatment with the anti- PD-1 immune checkpoint inhibitor pembrolizumab [20,21,22]. Patients in the KEYNOTE-001 trial with a PD-L1 score of ≥50% who had not received prior treatment, when treated with 2 or 10 mg/kg of pembrolizumab every 2–3 weeks, showed improved median survival (22.1 months vs. 15.4 months) over those who had received prior treatment [40].

### 4.1. Squamous Cell and Adenocarcinoma

Previous studies have examined the expression of PD-L1 in cases of NSCLC as a whole, but few have performed subgroup analysis with large samples to delineate any potential differences between the two main types of NSCLC, SCC and adenocarcinoma (AC). Shi and colleagues performed such an analysis using both immunohistochemistry and in situ hybridization to evaluate for PD-L1 expression in a cohort of 133 AC cases and 83 SCC cases. They revealed greater expression rates of PD-L1 in SCC when compared to AC cases (29% vs. 13.5%, respectively) [44].

Pawelczyk et al. performed a similar analysis in a cohort of 866 NSCLC samples. For the SCC subgroup, they discovered a mean expression level of PD-L1 of 47%. When divided into tiers of expression levels, it was further revealed that 64% of SCC cases displayed low expression levels (<1%), 24.9% displayed moderate expression levels (1–49%), and 11.3% displayed high expression levels (≥50%) [42]. Similar results were obtained in the AC subgroup. The average levels of PD-L1 expression were found to be 41% in these cases. Once divided into tiers based on the degree of PD-L1 expression, it was revealed that 69.2% of AC cases displayed low expression levels (<1%), 20.3% displayed moderate expression levels (1–49%), and 10.4% displayed high expression levels (≥50%) [42]. A positive correlation was noted between the degree of PD-L1 expression and the malignancy grading scheme for both SCC and AC cases, indicating that those in more advanced stages of disease experience greater benefit from targeted PD-L1 therapy [52].

### 4.2. KRAS Mutant Adenocarcinoma

Lung adenocarcinoma, the most common NSCLC subtype, is often impacted by oncogenic mutations. Up to 25–30% of adenocarcinoma cases have associated KRAS mutations. These mutations are not sensitive to current anti-EGFR therapies and thus require novel interventions to be directly targeted [53]. Compared to traditional chemotherapy, greater therapeutic success has been achieved with anti-PD-L1 therapy in those patients with KRAS mutations as shown in the OAK and Checkmate 057 trials especially in combination in checkpoint inhibitors [48,54]. Falk et al. assessed the distribution of PD-L1 expression of various KRAS mutants in cases of AC, and when compared to wild-type AC (6% expression), KRAS mutants were found to express much greater levels of PD-L1. Importantly, the specific subset of the KRAS mutation appeared to significantly impact the degree of PD-L1 expression, as G12C and G12V variants expressed the highest levels at 8% and 12.9%, respectively [55]. Understanding the etiology of the mutations is at the heart of predicting the significance of this biomarker, however. In NSCLC adenocarcinoma, KRAS G12C, G12V, and G12D are the most common KRAS alterations observed, seen in 40.5%, 19.8%, and 14.7% of KRAS mutated adenocarcinoma, respectively [56], where these 3 variants account for over 75% of cases. Recent work demonstrated the causal relationship between genotoxins, where the observed global C>A alteration seen in smoking resulted in G12C and G12V, whereas global C>T changes responsible for much of the observed KRAS G12D is linked to a clock-like, or old-age signature, particularly specific to men [56]. Thus, the G12C and G12V alterations that are especially enriched in lung cancer are most commonly the result of smoking induced toxicity, rather than a single strong driving mutation such as ALK or EGFR. As such these variants are more likely to have higher TMB and PD-L1 expression, as there is more neoantigen and immune activation. Adding to the emerging understanding between specific alterations and histology, *KRAS* G12D is the most common variant observed in colorectal cancer and is also the most common variant observed in KRAS mutant adenocarcinoma with enteric differentiation where IHC profiles can be indistinguishable. Neither have good responses to immunotherapy regardless of PD-L1 expression [57].

A pooled analysis of over 5000 patients performed by Liu et al. revealed similar findings; that KRAS mutant tumors were more likely to be positive for PD-L1 than wild-type tumors. Additionally, the mutational status correlated with elevated levels of inflammatory markers and heightened tumor immunogenicity. These characteristics surprisingly resulted in a significantly increased overall response rate to anti-PD-L1 therapy and a subsequent prolongation of overall survival in the KRAS mutant subsets when compared to wild-type cancers [58]. These findings could be attributed to the greater neoantigen production, and thus immunogenic response, from the elevated tumor mutational burden in this mutational subset. Regardless, these findings overwhelmingly highlight the benefit KRAS mutant AC can receive from targeting PD1-PD-L1 pathway [58].

Although rare, the definite percentage of PD-L1 expression levels in KRAS mutant squamous cell carcinoma needs to be explored. KRAS mutant NSCLC constitutes one-third of NSCLC but has remained resistant to targeted drugs until recently. Two agents targeting KRAS G12C; sotorasib and adagrisib has obtained accelerated approval and breakthrough therapy designation, respectively, from FDA for use in 2nd line setting.

### 4.3. Adenosquamous Carcinoma

The adenosquamous (ASC) variant of NSCLC is a small subtype that accounts for <4% of all cases of NSCLC [59]. As its name implies, its histological features combine aspects of both AC and SCC. Despite the small percentage of cases, it accounts for, ASC carries one of the worst prognoses of NSCLC variants. This appears to be due to its inclination for chemotherapeutic resistance and increased likelihood of metastasis compared to other NSCLC variants [60]. Given these challenges, studies have investigated alternate therapeutic routes and found success with EGFR-TKIs in those with advanced ASC harboring EGFR alterations [61]. These promising results have underscored the need to further investigate targeted and immune-centered approaches for those diagnosed with ASC without a driver mutation.

Given the previously discussed success achieved in the KEYNOTE trials with pembrolizumab in treating NSCLC variants that overexpressed PD-L1, researchers have shifted their focus to other NSCLC variants that may be susceptible targets for this immune-centered therapy. In the detailed subgroup analysis of NSCLC variants and their PD-L1 expression status performed by Pawelczyk and colleagues, the expression levels of PD-L1 in adenosquamous carcinoma were documented on the lower end of the spectrum, with 78.1% of cases displaying low levels (<1%) and only 3.1% reaching the threshold of ≥50% to qualify for high expression [42]. In addition to the previously analyzed AC and SCC cases, Shi et al. used both immunohistochemistry and in situ hybridization to evaluate PD-L1 expression in a cohort of 87 ASC cases, which revealed expression levels of 39% and 37%, respectively. When each tissue type was investigated individually, the glandular component was found to stain proportionally less than the squamous component (11% and 39%, respectively) [44]. This indicates that further subcategorization of ASC into its dominant variant may prove beneficial for guiding targeted immune therapy, as previous clinical trials have shown improved response rates and progression-free survival in cases exhibiting squamous histology treated with PD-L1 targeted immunotherapy [62].

Previous studies have also revealed tumor mutation burden (TMB) as a promising marker for predicting survival following immune therapy [63]. Cheng et al. built upon these studies and analyzed the TMB in a cohort of ASC cases. This data was then compared to survival outcomes and revealed a significant correlation between TMB and overall survival, thus indicating a potential benefit for immune therapy in patients with ASC who have a higher TMB [63]. Of note, both Shi et al. and Cheng et al. revealed increased occurrences of EGFR mutations in ASC cases (57% and 62%, respectively), indicating a potential benefit of targeting EGFR in addition to anti-PD-L1 therapy [44,64].

### 4.4. Large Cell Carcinoma

The previously discussed NSCLC subgroup analysis performed by Pawelczyk et al. revealed the highest mean levels of PD-L1 expression (57%) in large cell carcinoma cases. Further subdivision revealed that 51.6% of LCC cases displayed low expression levels (<1%), 38.7% displayed moderate expression levels (1–49%), and 9.7% displayed high expression levels (≥50%) [42].

### 4.5. Small Cell Carcinoma

Small cell lung cancer (SCLC) has been hypothesized to have immunogenic aspects due to its high somatic mutation rates and associated paraneoplastic syndromes [65]. These characteristics could make SCLC a viable target for immune checkpoint inhibitor (ICI) therapy given the array of neoantigens capable of inducing an anti-tumor response. Thus, clinical trials implementing ICI therapy in cases of SCLC have been pursued and shown modest increases in progression free survival and overall survival, but additional, specific biomarkers such as PD-L1 are needed to focus on patients who will receive the most therapeutic benefit.

Investigating the percentage of SCLC patients with PD-L1 over-expression has proved challenging. Much remains unknown and studies have reported controversial findings. In a small retrospective study, tumor cell PD-L1 positivity defined as >1% was lower than that seen in NSCLC, although 18.5% of stromal cells were positive. Contradictory to Schultheis et al., 82 of 99 cases (82.8%) of SCLC showed positive cell surface/membranous staining in >5% of tumor cells [25]. Hellmann, et al., demonstrated that 18% of SCLC had PD-L1 expression of >1% [35]. Yu and colleagues reported similar numbers for a cohort of 142 SCLC patients with >1% staining positivity in 19.7% of cases. Of note, tumor-associated lymphocytes and macrophages that stained >1% for PD-L1 expression were reported in even greater numbers in 41.5% of cases [66]. In the recent study where pembrolizumab monotherapy did not improve progression-free survival, except in the sub-group of SCLC patients with high PD-L1 expression at the stromal interface, only 1 PD-L1+ case out of 35 was found when using DAKO 22C3, which was assessed on tumor cells [52]. The presence of PD-L1+ tumor-associated macrophages (TAMs) and PD-1+ TILs indicates that the pathway may be activated in 20–35% of cases where PD-1 is expressed on CD4+ and CD8+ T-lymphocytes upon antigen receptor signaling [41,43].

Given this heterogeneity of results in the literature, Acheampong and colleagues performed a meta-analysis of PD-L1 expression that covered 2792 patients and displayed an estimated pooled prevalence of PD-L1 expression in cases of SCLC to be 26% with an interstudy range of PD-L1 expression of 83%. This analysis also revealed that expression of PD-L1 was shown to have a positive effect on overall survival in those diagnosed with SCLC, a contrast to data on high PD-L1 expression in NSCLC cases which has shown a shorter overall survival in this specific subset of patients [67,68]. Despite the proportionally lower percentages of PD-L1 expression reported in the literature for cases of SCLC, important clinical correlations have been revealed in these studies. Of particular importance is the association of therapeutic response and levels of tumor-infiltrating cells. While most studies have demonstrated <50% PD-L1 expression in cases of SCLC, the majority of PD-L1 expression in these SCLC cases occurs on the infiltrating immune cells rather than the tumor cells themselves. Additionally, high expression of PD-L1 on these immune cells has shown improved clinical outcomes in those diagnosed with SCLC [43].

The previously discussed variability seen in the literature can be attributed to the different assays and antibodies used when staining for PD-L1, as each test has unique sensitivities and specificities. Additionally, the variance in sample collecting method (core needle biopsy vs. resection) can also attribute to the reportable differences. Given the controversy in the literature and the potentially significant impact ICI therapy can have on those with SCLC, further longitudinal studies are needed to assess for additional biomarkers and the efficacy of ICI in SCLC cases.

### 4.6. Sarcomatoid Carcinoma

Sarcomatoid carcinoma (SC) of the lung is a highly aggressive type of NSCLC with sarcoma-like differentiation. The SC often portends a poor prognosis due to its early metastasis and resistance to platinum-based chemotherapy. The immunohistochemistry for SC for PD-L1 expression by using the PD-L1/CD274 (SP142) antibody was investigated by Sharma et al. The positive cases were defined by >1% PD-L1 expression in the tumor cells. The results of their study demonstrated that PD-L1 expression was reported in 75% of the cases. In this study, 78% of cases expressed PD-L1 ≥50%, and 22% of the positive cases expressed 1–49% of PD-L1 staining. The subset of cases with PD-L1 ≥50% expression revealed MET mutation [69].

Domblides et al. further investigated PD-L1 expression and the associated response to ICI therapy. Immunohistochemistry for SC for PD-L1 expression was performed by using a clone SP263 antibody. This assay revealed a median PD-L1 expression of 70%, with 94.7% of samples surpassing the positive staining cutoff of 5%. Of the samples collected, 50% also manifested a KRAS mutation. The expression of PD-L1 was noted to be greater in patients that responded to ICI therapy, as the objective response rate was 58.8% higher for positive cases. TMB burden was also assessed and revealed that all those patients with a higher TMB (>10 mut/Mb) displayed PD-L1 expression (median 70%) and associated TP53 mutations, with 43% also displaying a TP53/KRAS co-mutation. Additionally, those who responded better to ICI therapy, and thus had improved survival, also displayed a higher TMB. The overall results of this study revealed significant efficacy of ICI therapy in cases of SC, with a reported objective response rate of 40.5% and overall survival of 12.7 months. Comparing this data to previously reported chemotherapeutic interventions, this study revealed an absolute minimum improvement in the objective response rate and overall survival of 24% and 5 months, respectively [70]. Therefore, SC patients are presumably good candidates for ICI therapy.

### 4.7. Large Cell Neuroendocrine Carcinoma

Historically, PD-L1 expression in patients with large cell neuroendocrine carcinoma (LCNEC) has been reported at lower rates (0–25%), or even been negative, when compared to other types of NSCLC. The rarity of this disease and the difficulty in collecting adequate sample sizes have made for sparse data on the association between the efficacy of anti-PD-1 therapy and various mutations seen in cases of LCNEC. Additionally, reliable predictors regarding the efficacy of anti-PD-1 therapy are lacking in this specific demographic [71]. Thus, investigations are underway to determine biomarkers that accurately depict a therapeutic response in cases of LCNEC.

Shirasawa and colleagues detailed a cohort of eleven patients diagnosed with LCNEC who received anti-PD-1 therapy. PD-L1 expression was detected in 9% of these cases with a low staining proportion of 5%. While the PD-L1-positive tumors responded to anti-PD-1 therapy, this study also revealed a unique finding in which those with PD-L1-negative tumors also responded well to anti-PD-1 therapy (objective response rate and progression free survival of 40% and 4.4 months, respectively). The density of tumoral CD8-positive tumor-infiltrating lymphocytes (TILs) was also investigated as a potential marker of response to anti-PD-1 therapy, and it was found that those with a high density of TILs responded significantly better to treatment (progression free survival: 12.9 months) than those with a low density of TILs (progression free survival: 1.3 months) [72]. Thus, given the rarity of this tumor type and the therapeutic response seen in this cohort of patients regardless of PD-L1 expression, additional biomarkers for targeted interventions should be explored in cases of LCNEC.

### 4.8. Biomarkers Associated with Immune Checkpoint Inhibitors (ICI) Sensitivity and Resistance

TMB as a biomarker of sensitivity to immunotherapy has fallen out of favor in the most recent updates of NCCN guidelines (NCCN, NSCLC v3.2022), with PD-L1 IHC expressing, patient status, extent of disease, and tumor histology guiding immunotherapy recommendations. Emerging evidence has revealed numerous biomarkers that can be both indications of ICI sensitivity, as well as ICI resistance and even contraindication. From a molecular standpoint, immunotherapy relies on the PD1/PDL1 pathway and neoantigen presentation pathways. Amplification or activation of components of this pathway are predicted to increase sensitivity, whereas loss-of-function alterations or deletion in pathway steps will reduce sensitivity or even promote immunotherapy resistance. Briefly, most markers indicating sensitivity are linked to the underlying ability to promote PD-L1 expression pathway (JAK/STAT, IL7R) or the ability of the tumor create (KMT2C, TP53) or mask neoantigens (B2M, PTEN, CDKN2A, KEAP1) [73,74,75,76,77,78,79].

Additionally, contraindications are based on observations of toxicity risk or tumor hyperprogression. The most common contraindications relevant to lung cancer therapy have been classic activating mutations in EGFR, where prior TKI use has been linked to a long-lasting increased risk of severe and almost always fatal pneumonitis, requiring permanent discontinuation of immunotherapy, with the risk increasing with additionally therapeutic modalities such as radiotherapy [80,81]). Importantly, MDM2 amplification in solid tumors has been associated with the paradoxical tumor hyper-progression seen in patients who receive PD-1/PD-L1 checkpoint blockade [82,83]. A recent study of ICI with or without MDM2 amplifications concluded that ICI therapy was strongly contraindicated in MDM2 amplified tumors with significantly reduced survival time in the ICI cohort (*n* = 1105, *p* = 0.0018), whereas no significance was observed in the non-ICI cohort (*n* = 2285) when comparing MDM2 amplified and non-amplified tumors. Notably, the worst outcomes observed in patients with TMB high/MDM2 amplified tumors (*p* < 0.0001) [84]. Recent evidence has also implicated KEAP1 alterations, rather than the previously suspected STK11, in reduced sensitivity to immunotherapy, especially when co-mutated with KRAS [85,86,87]). KEAP1 alterations were seen in ~3% across 40,000 distinct cancer types in a pan-cancer study, with the highest prevalence seen in NSCLC (15.8%). Across cancer types with KEAP1 alterations, TMB is significantly higher (10 vs. 4 muts/Mb, *p* < 0.0001) and OS is significantly shorter (39 vs. 109 months, *p* < 0.0001) [88]). The prognostic value of KEAP1 alterations was found in early-stage (*p* = 0.0099) and associated with markedly inferior DFS in early-stage cancers (*p* = 0.0009) [88]. Of note, copy number alterations (especially deep deletion or arm-level deletions) of KEAP1 at 19p13 were associated with substantially lower immune infiltrates in most cancer types NSCLC [88]. A study of the impact of STK11 and/or KEAP1 mutation on benefit to immunotherapy in KRAS mutant NSCLC found KEAP1 co-mutation had shorter OS (*p* = 0.006), while STK11 co-mutation did not have an impact on OS (*p* = 0.3), with authors concluding, KEAP1 was a predictive factor in metastatic KRAS-mutated NSCLC, treated with immunotherapy [85].

The association between KRAS and PD-L1 is important to discuss in regard to NSCLC, as these mutations often coexist and can affect tumor characteristics and therapeutic responses. A systematic meta-analysis demonstrated that patients with KRAS mutations were shown to have clinical benefits when treated with anti-PD-1/PD-L1 immunotherapy. These studies also revealed that KRAS mutations are associated with better patient responses to PD-L1 inhibitors, which has been attributed to the presence of an inflammatory tumor microenvironment and heightened tumor immunogenicity. These environmental characteristics are specifically due to an increased ratio of PD-L1 and CD8+ tumor-infiltrating lymphocytes (TILs) and an increased TMB [58]. When clinical trials have been divided into subgroups and analyzed, KRAS mutant patients, have also been shown to be more sensitive to PD-1/PD-L1 inhibitors when compared to wild-type patients [89].

In a cohort of 2880 KRAS mutant NSCLC patients analyzed by Yang et al., PD-L1 expression levels were found to range from 12.82% (Q61 mutation) to 35.21% (G13 mutation) [90]. The G13 mutants, specifically, had a high tumor proportion score (>50% PD-L1 expression) with 46% of patients passing this threshold. These KRAS mutants also discovered a positive correlation between tumor mutational burden (TMB) and PD-L1 expression status. Multivariate analysis revealed the presence of a KRAS mutation as an independent predictor for the expression of PD-L1 on immune cells, and an increased propensity for the epithelial to mesenchymal transition (EMT) was also associated with a higher expression of PD-L1 [91]. Concomitant TP-53 mutations were also found to correlate with higher expression of PD-L1 and when combined with KRAS mutations, these led to higher levels of immune markers, inflammatory markers, and improved recurrence-free survival, ultimately suggesting a more invasive phenotype with a better response to therapy [92]. Similarly, when high mutational rates are present, this correlates with enhanced immunogenicity, indicating an elevated sensitivity to immune checkpoint blockage [91].

The targeting of PD-L1 as a therapeutic intervention for NSCLC has become a popular topic in both the research and clinical realms. The vast majority of current therapies aimed at PD-L1 are monoclonal antibodies, which have been shown to have clear advantages and disadvantages. In terms of pharmacodynamics, the structure of these antibodies allows for improved efficacy and specificity when targeting PD-L1. However, this structural advantage also proves a hindrance in regard to the pharmacokinetics involved, as these therapies have been shown to have poor oral bioavailability, a long half-life, and, ultimately, an extensive side effect profile [93]. Despite the potential downsides of these therapies, however, clinical studies such as KEYNOTE-024, KEYNOTE-042, and IMpower 110 have repeatedly shown a clear survival benefit when implementing PD-L1 therapies as compared to platinum-based chemotherapy [54]. Additional studies investigating combination therapies including PD-L1/platinum chemotherapies and PD-L1/CTLA-4 therapies (CheckMate 227 trial) have also shown survival benefits [92,94].

Despite the progress achieved with immune checkpoint therapy, many questions remain unanswered such as which therapy to implement as first-line and whether or not monotherapy is appropriate, how to properly evaluate biomarker status, and how to best combine immunotherapies with other agents efficaciously. Existing therapeutic plans allow for either monotherapy with PD-1/PD-L1 inhibitors or combination with platinum-based chemotherapy or other immune therapies, however, it remains unclear whether adding platinum-based chemotherapy to a pre-existing regimen of PD-1/PD-L1 inhibitors is beneficial in those cases where PD-L1 is strongly expressed. Thus, a phase III study is currently ongoing to address this question by comparing CBDCA/PEM/pembrolizumab to pembrolizumab in patients with a PD-L1 TPS ≥ 50% [95]. Additionally, as CTLA-4 inhibitors have gained notoriety as a standard treatment option for NSCLC in combination with PD-L1 inhibitors, questions have been raised regarding the comparative efficacy of PD-L1/CTLA-4 versus PD-L1/platinum regimens. Further studies are needed to address which combination strategy is most ideal for NSCLC patients and their relative PD-L1 expression status. In addition to these investigable treatment regimens, research must continue into evaluating more predictive biomarkers to better identify eligible patients and optimize their outcomes. Resistance to therapy has arisen as a final focus for future therapeutic endeavors, underscoring the importance of understanding how these resistances arise and developing mechanisms to overcome them [95].

### 4.9. Ongoing Clinical Trials

There are currently 148 active clinical trials investigating PD-L1 therapies in the setting of lung cancer. Of these studies, 120 are focused on NSCLC as a broad category. Small cell is the focus of 11 current studies, 5 of which are combination studies also involving NSCLC. Five studies solely focusing on one of the NSCLC variants were reported, with three for SCC and two for adenocarcinoma. Sarcomatoid cancer was the focus of one study, and the five remaining studies did not specify the type of lung cancer they were targeting. (https://www.clinicaltrials.gov/) (accessed on 15 June 2022).

### 4.10. Limitations

An important limitation of our study is that the queried databases/sites do not represent all published articles. These unassessed studies, some of which may have been published in other languages might show different results and conclusion which we did not include in our study protocol. We performed three internal contributor technique for validation of the study. However, our study was not externally validated.

## 5. Conclusions

Among the studies included in this analysis, there was no standard cut-off value used for dichotomization of results into PD-L1 positive and negative, making comparisons between cancer subtypes difficult. A value of >5% stained cells was frequently used, with or without an additional measure of intensity. Given the therapeutic significance of the ≥50% cut-off, it would be beneficial for future studies to report both values.

Within the current analysis, there is no separation of data based on smoking status, disease stage, age, gender, or prior treatment. Future studies should explore the effect of these variables on the status of PD-L1 expression levels. Additionally, the level of expression in SCLC stromal cells should be investigated, with special attention paid to the effects on response to treatment.

Limited data were available for PD-L1 expression in SCLC, LCNEC, KRAS mutant adenocarcinoma, and other less common forms of NSCLC, making evaluation difficult. Future studies should explore the expression levels in these uncommon cancers, as well as the response to checkpoint inhibitors, mainly those that inhibit PD-1 and PD-L1.

## Figures and Tables

**Table 1:** PD-L1 expression in subtypes of lung cancer.

Lung Cancer Subtype	PD-L1 ≥ 50%	PD-L1 1–49%	PD-L1 >1%	PD-L1 <1%	References
All NSCLC	540/4063 (13.29%)	1356/3184 (42.59%)	1763/4761 (37.03%)	2912/4613 (63.13%)	
Squamous Cell Carcinoma	284/1766 (16.08%)	569/1189 (47.86%)	743/1810 (41.05%)	1067/1810 (58.95%)	[22,25,35,40,41]
Adenocarcinoma	179/1919 (9.33%)	712/1507 (47.25%)	826/2379 (34.72%)	1553/2379 (65.28%)	[22,25,35,40,41]
KRAS Mutant Adenocarcinoma	-	37/136 (27.21%)	-	-	[40]
Adenosquamous Carcinoma	-	21/54 (38.89%)	3/7 (42.86%)	4/7 (57.14%)	[42]
Large Cell Carcinoma	-	10/35 (28.75%)	31/88 (35.23%)	52/77 (67.53%)	[42]
Large Cell Neuroendocrine Carcinoma	-	-	1/23 (4.35%)	22/23 (95.65%)	[43]
Small Cell Lung Cancer	-	73/194 (37.63%)	-	-	[25]
Sarcomatoid carcinoma	78%	22%	-	-	[44]
